# *Cryptosporidium parvum* disrupts intestinal epithelial barrier in neonatal mice through downregulation of cell junction molecules

**DOI:** 10.1371/journal.pntd.0012212

**Published:** 2024-05-24

**Authors:** Chaowei Luo, Yanhua Xu, Jie Zhang, Qing Tian, Yaqiong Guo, Na Li, Yaoyu Feng, Rui Xu, Lihua Xiao

**Affiliations:** 1 State Key Laboratory for Animal Disease Control and Prevention, South China Agricultural University, Guangzhou, China; 2 Guangdong Laboratory for Lingnan Modern Agriculture, Center for Emerging and Zoonotic Diseases, College of Veterinary Medicine, South China Agricultural University, Guangzhou, China; National University of Singapore, SINGAPORE

## Abstract

**Background:**

*Cryptosporidium* spp. cause watery diarrhea in humans and animals, especially in infants and neonates. They parasitize the apical surface of the epithelial cells in the intestinal lumen. However, the pathogenesis of *Cryptosporidium*-induced diarrhea is not fully understood yet.

**Methodology/principal findings:**

In this study, we infected C57BL/6j neonatal mice with *C*. *parvum* IIa and IId subtypes, and examined oocyst burden, pathological changes, and intestinal epithelial permeability during the infection. In addition, transcriptomic analyses were used to study the mechanism of diarrhea induced by the *C*. *parvum* IId subtype. The neonatal mice were sensitive to both *C*. *parvum* IIa and IId infection, but the IId subtype caused a wide oocyst shedding window and maintained the high oocyst burden in the mice compared with the IIa subtype. In addition, the mice infected with *C*. *parvum* IId resulted in severe intestinal damage at the peak of infection, leading to increased permeability of the epithelial barrier. The KEGG, GO and GSEA analyses revealed that the downregulation of adherens junction and cell junction molecules at 11 dpi. Meanwhile, E-cadherin, which is associated with adherens junction, was reduced at the protein level in mouse ileum at peak and late infection.

**Conclusions/significance:**

*C*. *parvum* IId infection causes more severe pathological damage than *C*. *parvum* IIa infection in neonatal mice. Furthermore, the impairment of the epithelial barrier during *C*. *parvum* IId infection results from the downregulation of intestinal junction proteins.

## Author summary

Cryptosporidiosis is a leading cause of moderate to severe diarrhea in children under 2 years of age. However, the pathogenesis of *Cryptosporidium* infection is not well understood. In this study, we used neonatal mice and a virulent *C*. *parvum* IId subtype as an infection model to study the mechanism of diarrhea associated with cryptosporidiosis. The results showed that the *C*. *parvum* IId subtype caused intense infection and severe pathological changes. At the peak of infection, mice had reduced intestinal digestion and absorption, and increased intestinal permeability. Transcriptomic data indicated that multiple pathways were involved in regulation of *C*. *parvum* IId infection. In particular, the adherens junction and cell junction assembly were downregulated. This was further supported by the reduction of E-cadherin expression during both peak and recovery periods. These data suggest that neonatal mice infect with *C*. *parvum* experience extensive damage of the intestinal barrier, resulting in diarrhea at the peak of infection.

## Introduction

*Cryptosporidium* spp. are intracellular but extracytoplasmic parasites that invade the gastrointestinal epithelium of vertebrate hosts, leading to the development of cryptosporidiosis [1]. This disease primarily affects individuals with weak immune systems, including young children, newborn animals, and immunocompromised individuals. In low- and middle-income countries, cryptosporidiosis ranks as the second most common cause of childhood diarrhea and a major cause of death in children under the age of two [2,3]. In high-income countries, cryptosporidiosis is a major cause of foodborne and waterborne illnesses [4]. Among the over 20 *Cryptosporidium* species and genotypes have been detected in humans, *C*. *parvum* and *C*. *hominis* are responsible for over 90% of cryptosporidiosis cases in humans around the world [5]. In addition, *C*. *parvum* is a major cause of diarrhea in newborn calves and lambs worldwide [6]. There is currently no clinical vaccine for cryptosporidiosis. The only anti-*Cryptosporidium* drug approved by the U.S. Food and Drug Administration is nitazoxanide. However, it is mostly ineffective in treating cryptosporidiosis in both infants and immunocompromised patients [7,8].

Neonatal animals such as mice [9], calves and goats [10] are highly susceptible to *Cryptosporidium* infection. Among them, neonatal mice are susceptible to *Cryptosporidium* but gradually become resistant to parasite infection as they age [11]. This suggests that neonatal mice are a suitable model for studying the pathogenesis of diarrhea and host immune responses caused by *Cryptosporidium*. However, the mechanisms behind the severe diarrhea induced by *Cryptosporidium* spp. are not yet well understood.

Zoonotic cryptosporidiosis is a significant public health issue in high-income countries, and it is primarily caused by *C*. *parvum* IIa subtypes that are prevalent in dairy calves. However, the spread of *Cryptosporidium* in China differs significantly from that in other regions, with the main subtype identified being IId [12]. The pathogenesis about cryptosporidiosis has been primarily conducted with the IOWA strain of the *C*. *parvum* IIa subtype family [13]. In recent years, this strain has undergone significant changes in subtype identity, resulting in reduced virulence in mice. In contrast, several IId subtypes from China induce more intensive infection in mice and are more virulent than the IOWA strain [14].

The aim of this study was to investigate the mechanism of diarrhea in cryptosporidiosis using the virulent *C*. *parvum* IId subtype and a neonatal mouse infection model. Our results indicate that *C*. *parvum* IId causes intense infection in neonatal mice. We demonstrated the occurrence of intestinal barrier breakdown during infection. A comparison of the differential gene expression profiles indicated the occurrence of downregulation of adherens junction and cell junction assembly in *C*. *parvum* IId infected neonatal mice. This was accompanied by reduced expression of the key cell junction molecule, E-cadherin.

## Materials and methods

### Ethics statement

Animal studies on mice were approved by the Committee on the Ethics of Animal Use in Research, South China Agricultural University (No. 2021-D118). This study was performed in compliance with the Guide to the Care and Use of Laboratory Animals.

### *C*. *parvum* and mouse infection

The *C*. *parvum* IIa-waterborne (IIa) isolate, also known as the IOWA isolate by the supplier and belonging to the IIaA17G2R1 subtype, was purchased from Waterborne, Inc. (New Orleans, LA, United States). The *C*. *parvum* IIdA20G1-HLJ (IId) isolate was obtained from a dairy calf from the farm that experienced significant mortality due to outbreaks of cryptosporidiosis in Heilongjiang province of China. The oocyst isolates were passaged in GKO mice and purified as previously described [15]. Twelve C57BL/6j mice in the third trimester of gestation were sourced from the Institute of Laboratory Animal Science at the China Academy of Medical Sciences. The mice were randomly assigned to three groups, each consisting of four pregnant females. Each female gave birth to 6 to 9 nursing offspring. At four days of age, 30 pups were orally infected with 1×10^5^ *C*. *parvum* IIa oocysts per mouse, 31 pups were infected with 1×10^5^ *C*. *parvum* IIa oocysts per mouse, and 30 pups were given PBS as a control. The volume of the gavaged sample was 10 μl per pup, and the pups were returned to their mother’s cages throughout the entirety of the experimental period.

### Quantification of oocyst shedding and body weight

Oocyst excretion was measured by analyzing fecal samples using 18S-LC2 qPCR every three days starting from day 0. Before three weeks of age, fecal matter was collected by stimulating excretion from the anus. After this point, excretion occurred spontaneously. The states of the feces were divided into four categories based on the criteria used in previous study: 0, normal feces; 1, exceptionally loose feces; 2, loose yellow feces; and 3, liquid feces [16]. After fecal collection, DNA was extracted from 100 mg of fecal material using the FastDNA SPIN Kit for soil (MP Biomedicals, Santa Ana, CA, USA). Quantitative PCR was performed using the LightCycler 480 Instrument II (Roche, Basel, Switzerland) to target the *SSU* rRNA gene. Each 20 μl reaction contained 1 μl of fecal DNA, 500 nM primer solutions targeting *C*. *parvum* and 10 μl THUNDERBIRD SYBR qPCR Mix (Toyobo) [17]. The oocysts per gram of feces (OPG) were calculated using the qPCR Cq values obtained from the assay and a standard curve produced with fecal samples containing precise counts of *C*. *parvum* IIa oocysts. A result was considered negative for *C*. *parvum* if the log_10_(OPG) value was less than 2.5. To confirm the negative results, a collateral analysis of the *SSU* rRNA gene was conducted at regular intervals using nested PCR [17]. The body weights of these mice were recorded every three days from 3 days post infection (dpi) to 30 dpi. Based on the findings, the oocyst excretion curve was labeled for three periods: the early period (i.e. 3 dpi), peak period (i.e. 11 dpi), and late period (i.e. 30 dpi).

### Histological examination and evaluation by hematoxylin and eosin (H&E) staining

The C57BL/6j mice were subsequently euthanized and the ileum samples were collected at 3 dpi, 11 dpi and 30 dpi, with 4 mice in each group per time point. The collected samples were fixed in 4% paraformaldehyde for 24 hours, embedded in paraffin, and subsequently cut into 4 μm-thick sections. Hematoxylin and eosin staining was performed using conventional procedures by the Servicebio company. To evaluate the function of ileum mucosal barrier in mice, we measured the length of villi, depth of crypts, and crypt depth ratios. Additionally, we counted the number of parasites by observing microscopic parasites per 15 intestinal villi at three different infection periods. The stained slides were examined under a Zeiss Axioskop Mot 2 fluorescence microscope (Carl Zeiss, Oberkochen, Germany), and analyzed using ImageJ software. Histological pathology was evaluated based on the criteria used in previous study: crypto damage (0–4 score), severity of inflammation (0–3 score), and depth of injury (0–3 score) [18].

### Immunofluorescence staining and periodic acid-Schiff (PAS) staining

The 4 μM sections were dewaxed twice in xylene for 5 mins each time, followed by rehydration using gradient alcohol (100%, 90%, 80%, 70%) for 3 mins each. After washing the slide once with PBS, the tissue was circled using a waterproof pen. For immunofluorescence staining, Antigen repair solution was applied to the tissue at a temperature above 95°C for 10 mins. The tissue was then blocked with 10% goat serum for 2 hours, followed by incubation with the anti-*Cryptosporidium* antibody Sporo-Glo (Waterborne) at a ratio of 1:50 in 0.1% BSA-PBS for 1 hour. After rinsing with PBS, the slices were treated with the Slowfade Gold anti-fading agent containing DAPI reagent (Invitrogen) and covered with glass slides. For PAS staining, after rehydration and washes with ddH_2_O, the samples were incubated in periodic acid (Shyuanye, Shanghai, China) for 5 mins, washed 3 times with ddH_2_O, incubated in Schiff’s reagent (Shyuanye, Shanghai, China) for 15 mins and washed 10 mins with tap water. Then, the rest of the staining was followed by the steps of H&E staining described above. The stained slides were examined under a Zeiss Axioskop Mot 2 fluorescence microscope (Carl Zeiss, Oberkochen, Germany), and the *C*. *parvum* counts were determined using ImageJ software.

### Permeability assay

The detection of mouse urine was carried out using the Fluorescein Isothiocyanate-dextran (FD4) permeability assay [19]. At each time point, four mice in each group were used for permeability assay. The specific steps for gavage according to body weight were followed with a dosage of 400 mg/kg. After 4 hours, the mouse’s bladder was stimulated to promote urine excretion, and the urine was collected. Fluorescence values were measured using an excitation light at 485 nm and an absorbance light at 528 nm.

### Characterization of intestinal epithelial cells junction using transmission electron microscopy (TEM)

To observe cell-cell junctions after *C*. *parvum* infection, the ileum samples were selected from a *C*. *parvum* IId infected group and control group at 3 dpi, 11 dpi, and 30 dpi. The ileum samples were fixed overnight at 4°C in 4% paraformaldehyde and 0.1% glutaraldehyde in PBS, washed with PBS, embedded in low-melting agarose and dehydrated in a gradient of ethanol at -20°C. The samples were then infiltrated with LR White acrylic resin (Sigma, St. Louis, MO, USA) for overnight at -20°C and polymerized with fresh LR White in gelatin capsules at -25°C for three days. Thin sections measuring 50 nm were obtained using a diamond knife from Leica (Wetzlar, Germany) and fixed onto nickel grids. The slices were viewed using a field emission transmission electron microscope (FEI Company, Hillsboro, OR, USA).

### RNA sequencing

Ileum samples were collected from both *C*. *parvum* IId infected mice and uninfected mice at 3, 11, and 30 dpi. Both the infected group and the control group contained 4 samples at each time point. Total RNA was extracted from ileum samples at three time points using Ambion Trizol Reagent (ThermoFisher, Rockford, IL, USA), following the manufacturer’s instructions. RNA purity and integrity were assessed by using the ND-1000 Nanodrop and the Agilent 2200 Tape Station. Quality control parameters included that A260/A280 ratio > 1.8, A260/A230 ratio > 2.0 and RNA integrity number value > 7.0 [20]. RNA-seq libraries were generated using the TruSeq RNA sample preparation kit (Illumina), and high-throughput sequencing was performed on the Illumina HiSeq 2500 system. Quality control on the raw data was performed using FastQC (v0.11.9, https://www.bioinformatics.babraham.ac.uk/projects/fastqc/) and Fastp (v0.23.1, https://github.com/OpenGene/fastp). The clean data was then aligned to the reference genome of mice (Ensembl GRCm39, https://asia.ensembl.org/index.html) by STAR (v2.7.2b, https://github.com/alexdobin/STAR), and expression was counted by RSEM (v1.3.1, https://github.com/deweylab/RSEM/).

### Bioinformatic analysis and gene set enrichment analysis (GSEA)

The samples were divided into three groups based on the sampling time: 3 dpi, 11 dpi, and 30 dpi. To assess reproducibility and experimental variation among biological replicates, the expression levels of genes were represented by fragments per kilobase per million mapped fragments (FPKM). The principal component analysis (PCA) of FPKM demonstrated the correlation among the samples. DESeq2 within R (v4.2.2, https://www.r-project.org/) was utilized to analyze differentially expressed genes (DEGs) with a significant threshold of padj ≤ 0.05 and log_2_(fold change) ≥ 1. The list of DEGs was subjected to Kyoto Encyclopedia of Genes and Genomes (KEGG) pathway enrichment analysis, and Gene Ontology (GO) term enrichment analysis using the ClusterProfiler package (v4.10.0). Gene Set Enrichment Analysis (GSEA) analysis was performed on all genes using the ClusterProfiler package (v4.10.0). Visualization was carried out with the GseaVis package (v0.0.9). The heatmap illustrating the DEGs was created using pheatmap (v1.0.12), while the remaining plots were visualized using the ggplot2, stringr, ggrepel, and dplyr R packages. To investigate the changing trend of host genes at different periods after *Cryptosporidium* infection, STEM (v1.3.13) was utilized to analyze the expression trends of DEGs at three timepoints. A maximum of 20 profiles were set to be output, and then GO and KEGG enrichment analysis was performed on the genes selected from the significantly enriched profiles to study the functions and major pathways of genes with the same expression pattern.

### Immunofluorescence staining of E-cadherin

The 4 μM sections were obtained using a cryostat microtome (Leica, Wetzlar, Germany) and placed onto glass slides. The paraffin sections underwent two rounds of dewaxing in xylene, followed by gradient alcohol treatment (100%, 90%, 80%, 70%) for 3 mins each. The sections were then washed with PBS and repaired with acid antigen repair solution at 100°C for 10 mins. The sections were treated with 10% goat serum at room temperature for 1 hour, then incubated with E-cadherin (Abmart, Berkeley Heights, NJ, USA) diluted in 1:100 for overnight at 4°C. Subsequently, the slices were incubated with Alexa Fluor 488 coupled goat anti-mouse IgG (ThermoFisher) diluted in 1:1000 and Sporo-Glo diluted in 1:50 at room temperature for 1 hour. The slides were rinsed with PBS and then treated with the Slowfade Gold anti-fading agent containing DAPI reagent (ThermoFisher) to the cover glass. The stained slides were examined under a Zeiss Axioskop Mot 2 fluorescence microscope (Carl Zeiss, Oberkochen, Germany). The images were processed using ZEN microscopy software (Carl Zeiss, Oberkochen, Germany).

### Real-time quantitative PCR

The cDNA synthesis was carried out with the PrimeScript reverse transcriptase reagent kit (Yisheng Biotechnology Shanghai Co., Ltd). Real-time quantitative PCR (RT-PCR) was performed using THUNDERBIRD SYBR qPCR Mix (Toyobo) on the LightCycler 480 Instrument II (Roche, Basel, Switzerland). The transcription levels of *cdh1* were measured by the 2^−ΔΔCt^ method. The primers used in this study were GAPDH as control (forward, 5’-GAAGGGCTCATGACCACAGT; reverse, 5’-TGCAGGGATGATGTTCTGGG) and *cdh1* (forward, 5’- CCCTGCCTCTGAATCCAACC; reverse, 5’- TGTCCCTGTTGGATTTGATCTGAA). The design of primers was developed based on the NCBI Primer-BLAST online tool (https://www.ncbi.nlm.nih.gov/tools/primer-blast/).

### Western blot

To determine the protein expression level of E-cadherin, the intestinal ileum tissue was extracted and lysed in 200 μl of RIPA buffer containing protease inhibitors (Beyotime, Shanghai, China) on the ice for 5 mins. Both the infected group and the control group contained 4 mouse samples at each time point. The lysed tissue was then homogenized using a tissue crusher and centrifuged at 15000 rpm, 4°C for 10 mins. The protein concentration was measured using the BCA method (Beyotime). Samples were resolved by SDS-PAGE using 50 μg of protein each and transferred onto nitrocellulose membranes. The membranes were blocked with 5% skimmed milk and then probed with E-cadherin (Abmart) diluted in 1:1000, followed by horseradish peroxidase labeled goat anti-rabbit IgG (H+L) diluted in 1:1000 (Beyotime). The membranes were analyzed using the Super Signal West Pico Chemiluminescent Substrate Kit (ThermoFisher) and were scanned on the UVP ChemStudio PLUS Imager (Analytik-jena).

### Statistical analysis

All statistical analyses were performed using the GraphPad Prism9 software. For mouse experiments, each infected group was compared to control group on individual days using two-way ANOVA tests. Statistical parameters for each experiment including test used, technical replicates (n) and *P* value are reported in the figure legends and associated with method details.

## Results

### Susceptibility of *C*. *parvum* IId infection in neonatal mice

**Fig 1:**
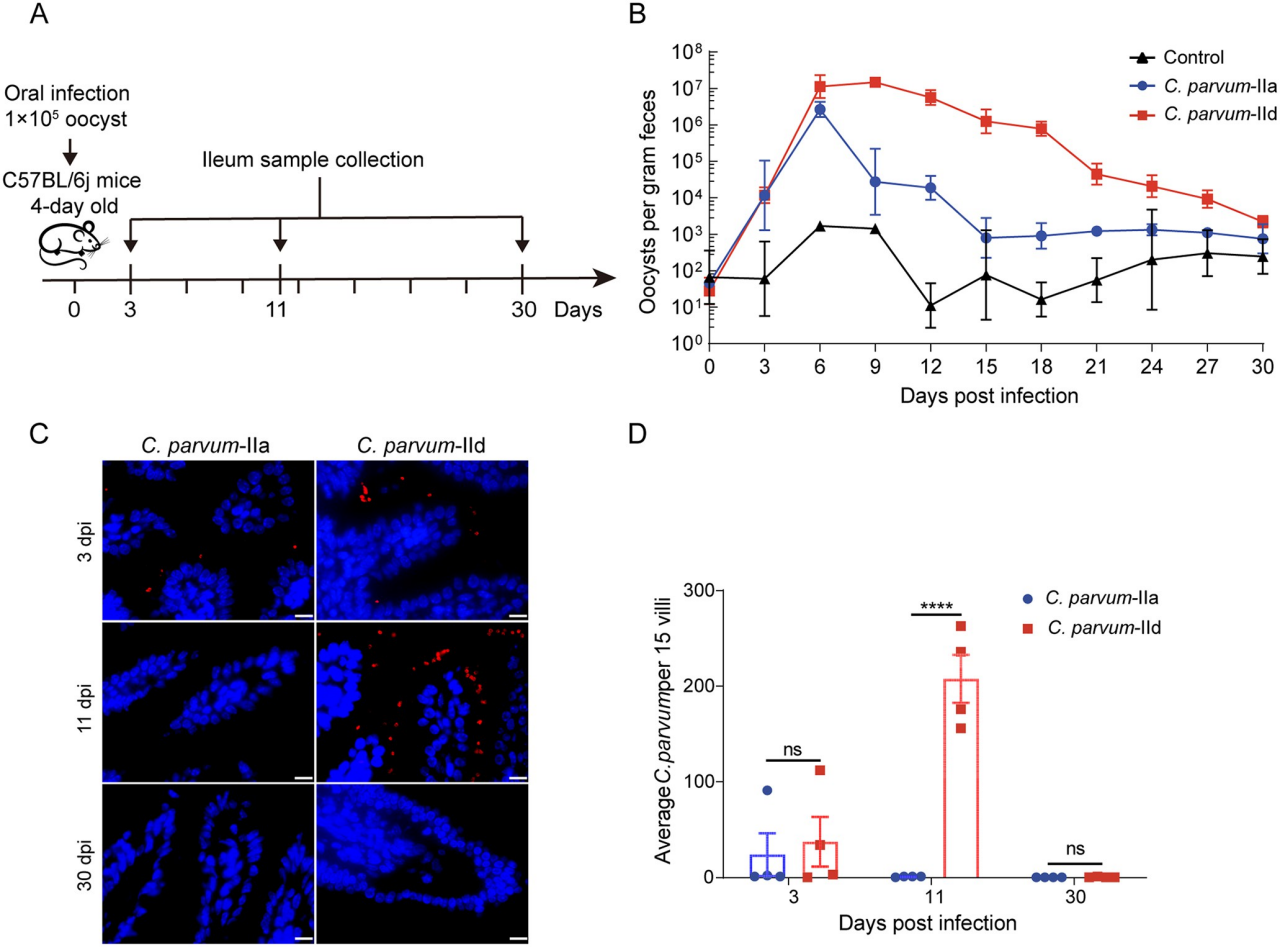
The neonatal mouse model of *C*. *parvum* IIa and *C*. *parvum* IId. (A) The 4-day-old C57BL/6j mice were infected with 1×10^5^ *C*. *parvum* IIa-Waterborne (IIa) or IIdA20G1-HLJ (IId) strain per mouse. Oocyst numbers were quantified from feces collected at intervals post infection. The ileum samples were collected at 3 days post infection (dpi), 11 dpi and 30 dpi for further analysis. (The direct link to the mouse cartoon: https://openclipart.org/detail/213632/mouse) (B) Oocyst shedding patterns of *C*. *parvum* IIa and IId in neonatal mice. (C) Immunofluorescence staining of *C*. *parvum* in mice ileum. Parasites were labeled with Sporo-Glo antibodies (red), and nuclei were stained with DAPI (blue). Scale bars = 10 μm. (D) The number of *C*. *parvum* per 15 intestinal villi at 3 dpi, 11 dpi and 30 dpi. All data were represented as mean ± S.D. Differences between *C*. *parvum* IIa and *C*. *parvum* IId were analyzed using two-way ANOVA following Sidak’s method. ****, *P* < 0.0001; ns, not significant.

To evaluate the susceptibility of neonatal mice to *C*. *parvum* infection, we infected twelve liters of mice and infected them with either the *C*. *parvum* IIa IOWA (IIa) or *C*. *parvum* IIdA20G1-HLJ (IId) strain, each litter contained 7 to 9 newborn mice. No evidence of *C*. *parvum* infection was detected in neonatal mice by nested PCR on the third day after birth. On the fourth day, each mouse was orally administered 1×10^5^ oocysts. Four litters were infected with each strain of *C*. *parvum*, while PBS was used as the control (Fig 1A). The burden of oocysts was measured in the feces of mice from 3 to 30 dpi using qPCR. Neonatal mice infected with *C*. *parvum* did not exhibit any significant changes in body weight compared to the control group (S1A Fig). The oocysts per gram of feces (OPG) in neonatal mice infected with *C*. *parvum* IId could reach over 1.5×10^7^, which was 5 times higher than the OPG value of 2.9×10^6^ in neonatal mice infected with *C*. *parvum* IIa. The peak shedding window for *C*. *parvum* IId was around 12 days (i.e. 6–18 dpi), which was much longer than the shedding window for *C*. *parvum* IIa, which only lasted 3 days (i.e. 6–9 dpi) (Fig 1B). The diarrhea disease severity scores reached peak levels at 6 dpi in mice infected with *C*. *parvum* IIa and at 9 dpi in mice infected with *C*. *parvum* IId (S1B Fig), which according to the oocyst shedding patterns. These findings indicate that neonatal mice are more susceptible to *C*. *parvum* IId infection than to IIa infection.

To further investigate the parasite load in the intestinal tissue, we used the immunofluorescence staining to visualize the parasite burden in the mouse ileum at 3, 11 and 30 dpi (Fig 1C). We compared the parasite burdens in different periods between the *C*. *parvum* IIa and *C*. *parvum* IId infected groups. No parasites were observed in the ileum of *C*. *parvum* IIa or IId infected mice at 30 dpi (Fig 1D), indicating that C57BL/6j mice were resistant to *C*. *parvum* infection after one month of age. Based on the oocyst shedding results, the number of parasitic infections in the *C*. *parvum* IId group was significantly higher than in the *C*. *parvum* IIa group at 11 dpi (Fig 1D) in ileum tissue. These findings suggest that *C*. *parvum* IId infection may cause more severe pathologies in neonatal mice than *C*. *parvum* IIa infection.

### *C*. *parvum* IId infection caused the intestinal damage in neonatal mice

**Fig 2:**
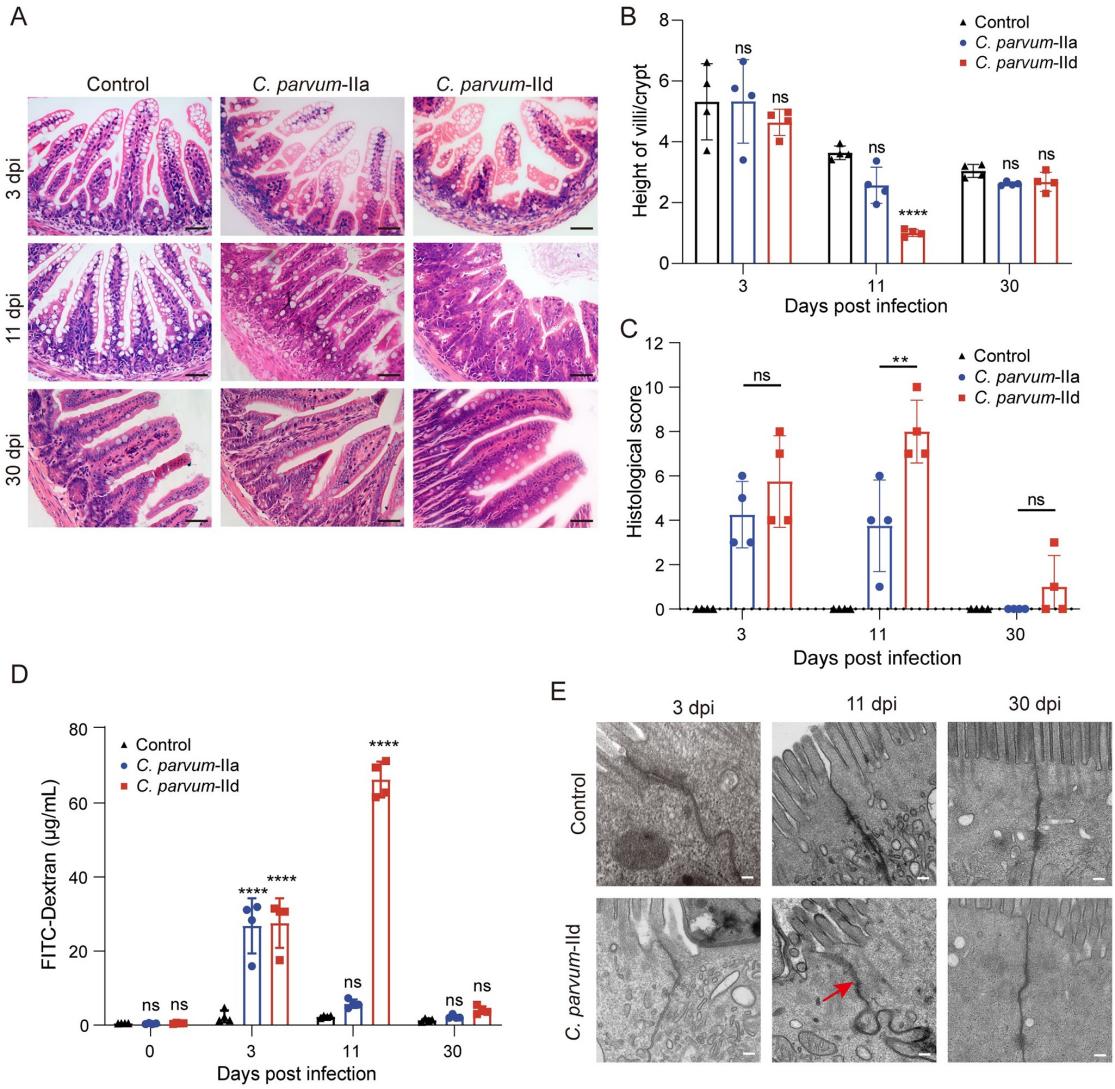
*C*. *parvum* infection led to intestinal damage in neonatal mice and *C*. *parvum* IId disruption of intestinal epithelial cell junction during the peak infection period. (A) Hematoxylin and eosin staining of the ileum of neonatal mice infected with *C*. *parvum* IIa, IId or uninfected control at indicated days post-infection. Scale bars = 40 μm. (B) The ratio of height of villi and crypt depth in the mouse ileum at indicated days post-infection. All data were represented as mean ± S.D. Differences between *C*. *parvum* IIa or IId and control were analyzed by two-way ANOVA following Dunnett’s method. ****, *P* < 0.0001 which *C*. *parvum* IId infected mice vs control at 11 dpi; ns, not significant. (C) Histological score of the mouse ileum at indicated days post-infection. All data were represented as mean ± S.D. Differences between *C*. *parvum* IIa and IId were analyzed by two-way ANOVA following Sidak’s method. **, *P* = 0.0036; ns, not significant. (D) Concentration of fluorescein isothiocyanate (FITC) dextran in urine of *C*. *parvum* IIa or IId infected or control mouse at indicated days post-infection. All data were represented as mean ± S.D. Differences between *C*. *parvum* IIa or IId mice vs control analyzed by two-way ANOVA following Dunnett’s method. ****, *P* < 0.0001 which *C*. *parvum* IIa or IId infected mice vs control; ns, not significant. (E) Transmission electron micrographs of intestinal epithelial cells in *C*. *parvum* IId infected or uninfected mice at indicated days post-infection. The red arrow indicated the cell adhesion structure. Scale bars = 200 nm.

To validate the changes in intestinal damage caused by *C*. *parvum* IIa or IId during the infection, mice ileum tissues were collected at 3, 11 and 30 dpi. Intestinal damage was detected through H&E staining. No intestinal damage was observed among the three groups of neonatal mice at 3 dpi and 30 dpi (Fig 2A). However, during peak infection, the length of intestinal villi in the ileum section of the *C*. *parvum* IId infection group was significantly shorter compared to the *C*. *parvum* IIa group, with an increased depth of crypt (Fig 2A). To further confirm our findings, we measured the ratio of villus length to crypt depth of the ileum. At 11 dpi, the *C*. *parvum* IId infected group showed a significantly lower ratio compared to the control group (Fig 2B). In addition, inflammatory cells, including lymphocytes and eosinophilic granulocytes, were observed in the mouse ileum through the whole period of *C*. *parvum* IId infection and the early stage of *C*. *parvum* IIa infection (S2A Fig). Furthermore, the histological damage was evaluated by scoring. The result demonstrated that the degree of damage in mice infected with *C*. *parvum* IId was significantly higher than that in mice infected with *C*. *parvum* IIa at 11 dpi (Fig 2C). To further compare the intestinal damage between *C*. *parvum* IIa infected mice and *C*. *parvum* IId infected mice, the PAS staining was employed to quantify the polysaccharides and mucins in the intestinal tissue. The results showed a reduction in the number of PAS-positive particles in the ileum of *C*. *parvum* IId infected mice at 11 dpi (S2B Fig). In total, the neonatal mice infected with *C*. *parvum* IId during the peak oocyst shedding period experienced a significant degree of intestinal damage, which may result in a weakened intestinal absorption capacity.

To verify the dysfunction of the intestinal epithelial mucosal barrier, we measured FD4 permeability in mouse urine at these three time periods. As shown in Fig 2D, both *C*. *parvum* IIa and IId infections led to increased FD4 permeability at 3 dpi. Additionally, *C*. *parvum* IId infection also showed increased permeability at 11 dpi. The FD4 permeability in infected groups almost returned to normal levels at 30 dpi (Fig 2D). Furthermore, transmission electron microscopy (TEM) was used to measure the intestinal permeability in mice infected with *C*. *parvum* IId during the peak period. The TEM analysis revealed clear morphological changes in cell junctions in mice infected with *C*. *parvum* IId at 11 dpi (Fig 2E). A close connection between cells was observed and no substantial morphological changes were found in either infected or control group at 3 dpi and 30 dpi. However, the cell junctions appeared diffused, and widened, and the fibrous filaments of adhesive connections vanished at 11 dpi (Fig 2E). These findings collectively demonstrated that *C*. *parvum* IId infection causes long-term diarrhea in neonatal mice following the substantial damage in mouse intestine.

### Differentially expressed genes in *C*. *parvum* IId infected and uninfected mice

**Fig 3:**
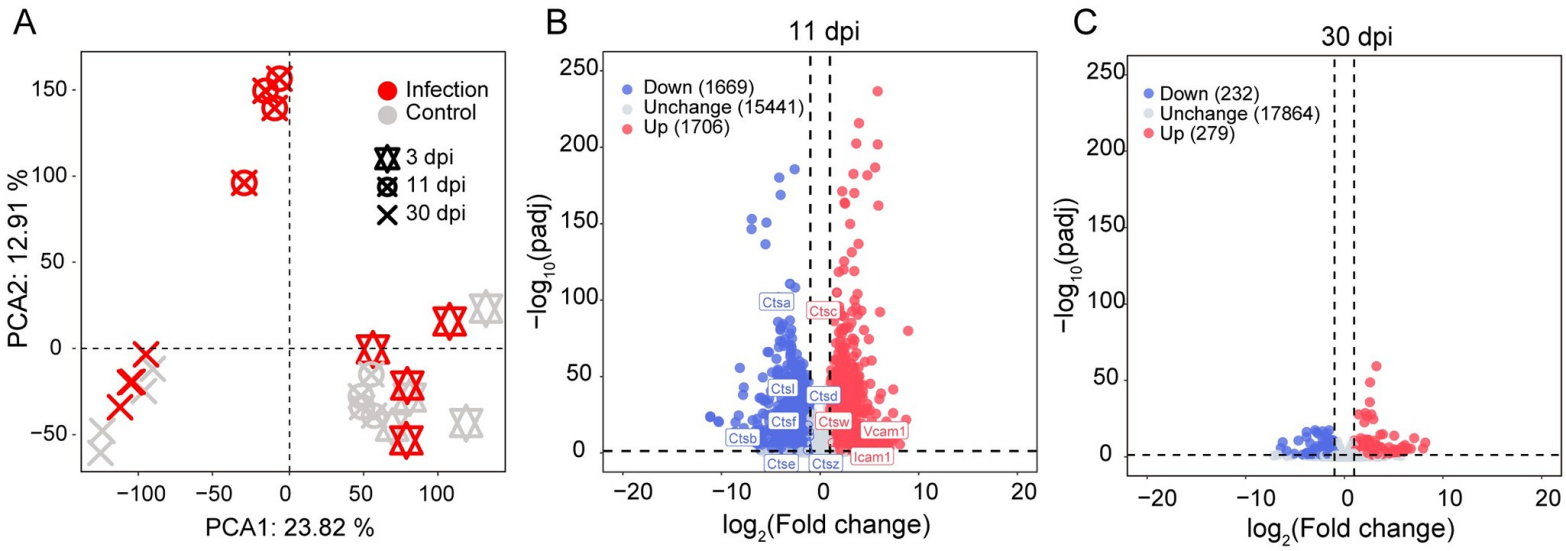
Principal component analysis and differentially expressed genes at 11 and 30 dpi. (A) The PCA analysis based on FPKM of all mouse genes in *C*. *parvum* IId infected or uninfected neonatal mice at indicated days post-infection. (B-C) Volcano plots of DEGs detected in mice infected with *C*. *parvum* IId or uninfected mice at 11 dpi (B) or 30 dpi (C). Up-regulated genes, the fold change of gene expression level in infected vs uninfected ≥ 2 and padj ≤ 0.05. Down-regulated genes, the fold change of gene expression level in infected vs uninfected ≥ -2 and padj ≤ 0.05. The others were considered unchanged. Some genes of interests were labeled.

Transcriptomic analysis was performed on neonatal mice infected with *C*. *parvum* IId, RNA was extracted from ileum tissues collected at 3 dpi, 11 dpi and 30 dpi and reverse transcribed into cDNA for RNA-seq analysis (Bioproject number is PRJNA:1072559). Approximately 200 million reads per sample were generated. After alignment, an average of 87.6% (3 dpi), 88.3% (11 dpi) and 88.5% (30 dpi) of high-quality reads were mapped against the reference *Mus musculus* genome. The correlation between samples from the infection and the control groups over the three periods was determined by principal-component analysis (PCA) based on host gene expression levels. The resulting PCA plot showed that the samples formed 3 major clusters. The infected and control groups at 3 dpi and 30 dpi were clustered separately. The uninfected samples at 11 dpi clustered with infected and uninfected samples at 30 dpi and were clearly different from the infection samples at 11 dpi (Fig 3A). The results indicate that mice did not experience significant effects during the early (3 dpi) and late stages (30 dpi) of infection with *C*. *parvum* IId. However, at the peak of infection (11 dpi), a notable difference was observed between the infected group and the control group.

Differentially expressed genes (DEGs) were detected in the infected and control groups at three time points based on the reads count. Volcano plots were used to illustrate the DEGs identified in the infected and control groups with at least a 2-fold change and a padj of less than 0.05. Only 39 DEGs were identified between the infected and control groups at 3 dpi, so further analysis was not performed. There were 3375 DEGs at 11 dpi, with 1706 up-regulated and 1669 down-regulated genes (Fig 3B). The genes labeled in Fig 3B belong to the immunoglobulin superfamily and are associated with cell adhesion molecules and calcium-dependent adhesion molecules. At 30 dpi, 511 DEGs were identified between the infected and control groups, with 279 genes upregulated and 232 genes down-regulated (Fig 3C).

### Enrichment analysis of GO functions and KEGG pathways

**Fig 4:**
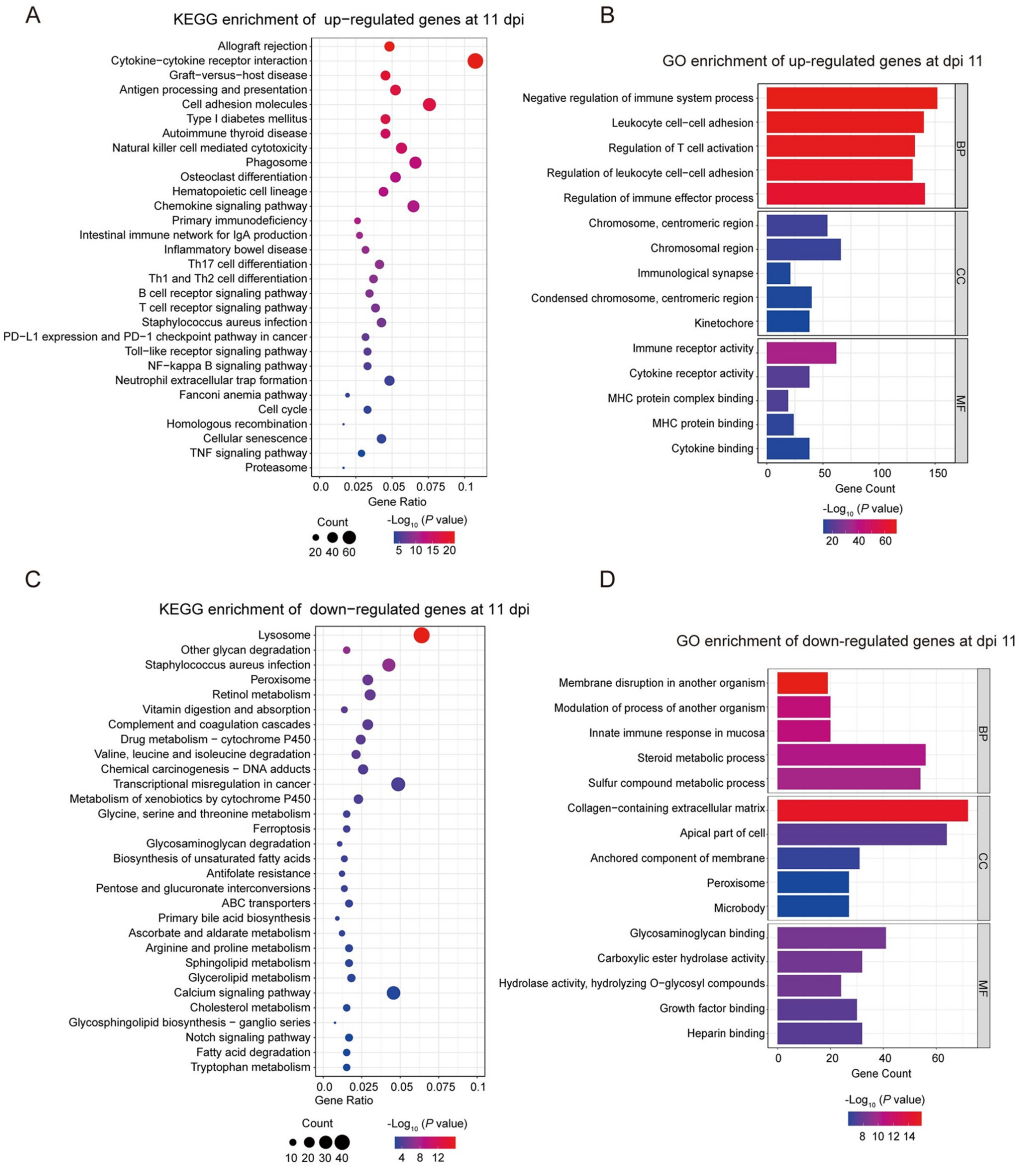
Analysis of the KEGG pathway and GO terms in *C*. *parvum* IId infected and uninfected mice at 11 dpi. (A) The KEGG enrichment of upregulated genes in *C*. *parvum* IId infected mice compared with uninfected mice at 11 dpi. The 1706 upregulated genes were analyzed for KEGG pathway. (B) The GO enrichment of upregulated genes in infected mice compared with uninfected mice at 11 dpi. (C) The KEGG enrichment of downregulated genes in *C*. *parvum* IId infected mice compared with control mice at 11 dpi. The 1669 downregulated genes were analyzed for KEGG pathway. (D) The GO enrichment of downregulated genes in infected mice compared with control mice at 11 dpi. The x-axis indicated the number of genes contained in each term. Terms in each category are ordered by *P* value.

The transcriptomic responses of the infected mice were most significant at 11 dpi and 30 dpi. Therefore, we focused on comparing the RNA-seq data at these two time points. The KEGG enrichment analysis at 11 dpi revealed that 1706 genes were upregulated and significantly enriched in 93 pathways. The most significant up-regulated pathways were allograft rejection, cytokine-cytokine receptor interaction, graft-versus-host disease, antigen processing and presentation, and cell adhesion molecules. (Fig 4A). Upon further analysis, it was found that the functional classification and enrichment analysis of 1706 up-regulated genes at 11 dpi showed significantly enriched in 1397 GO terms (*P* < 0.05). Among them, 1220 GO terms belonged to the biological processes (BP) category (e.g. negative regulation of immune system process, leukocyte cell-cell adhesion), 71 terms belonged to cell component (CC) category (e.g. chromosome, centromeric region), and 106 terms belonged to molecular function (MF) category (e.g. immune receptor activity, cytokine receptor activity) (Fig 4B). Based on the KEGG and GKO term analysis, the DEGs which upregulated at 11 dpi were involved in Th1 and Th2 responses, indicating that the mice were experiencing a strong inflammatory condition.

The KEGG enrichment analysis revealed that 1669 downregulated genes were significantly enriched in 59 pathways at 11 dpi (Fig 4C). The most prominent downregulated pathways were lysosome, other glycan degradation, staphylococcus aureus infection, peroxisome and retinol metabolism (Fig 4C). Furthermore, the GO enrichment analysis of downregulated genes at 11 dpi showed that 550 GO terms were significantly enriched in the 1669 downregulated genes. Of the total number of terms, 377 GO terms were classified under the BP category, including innate immune response in mucosa. The CC category had 59 GO terms, including collagen-containing extracellular matrix and apical part of cells. The MF category had 114 GO terms, such as glycosaminoglycan binding (Fig 4D). The DEGs downregulated in peroxisome and metabolism pathways indicate that the intestinal cells were undergoing the autophagy process.

Additionally, we performed the KEGG analysis on the DEGs during the late infection period (30 dpi). The analysis revealed a significant upregulation of multiple pathways during the late stage of infection (S3A Fig), which was also significantly upregulated at the peak stage (11 dpi). In contrast, the downregulated genes were seen to be significantly enriched in staphylococcus aureus infection, NOD-like receptor signaling, and transcriptional mis-regulation in cancer pathways (S3B Fig). Furthermore, the analysis of the primary expression modules utilized in STEM was illustrated in S4 Fig. The conclusions drawn are supported by the results of the STEM analysis of the *C*. *parvum* IId dataset. These data suggest that *C*. *parvum* IId infection induces cancerous growth in parasitic cells even after the parasite has been eliminated indicating that parasite infection may have a long-term detrimental effect.

### Gene set enrichment analysis of cell adhesion related genes at the peak infection

**Fig 5:**
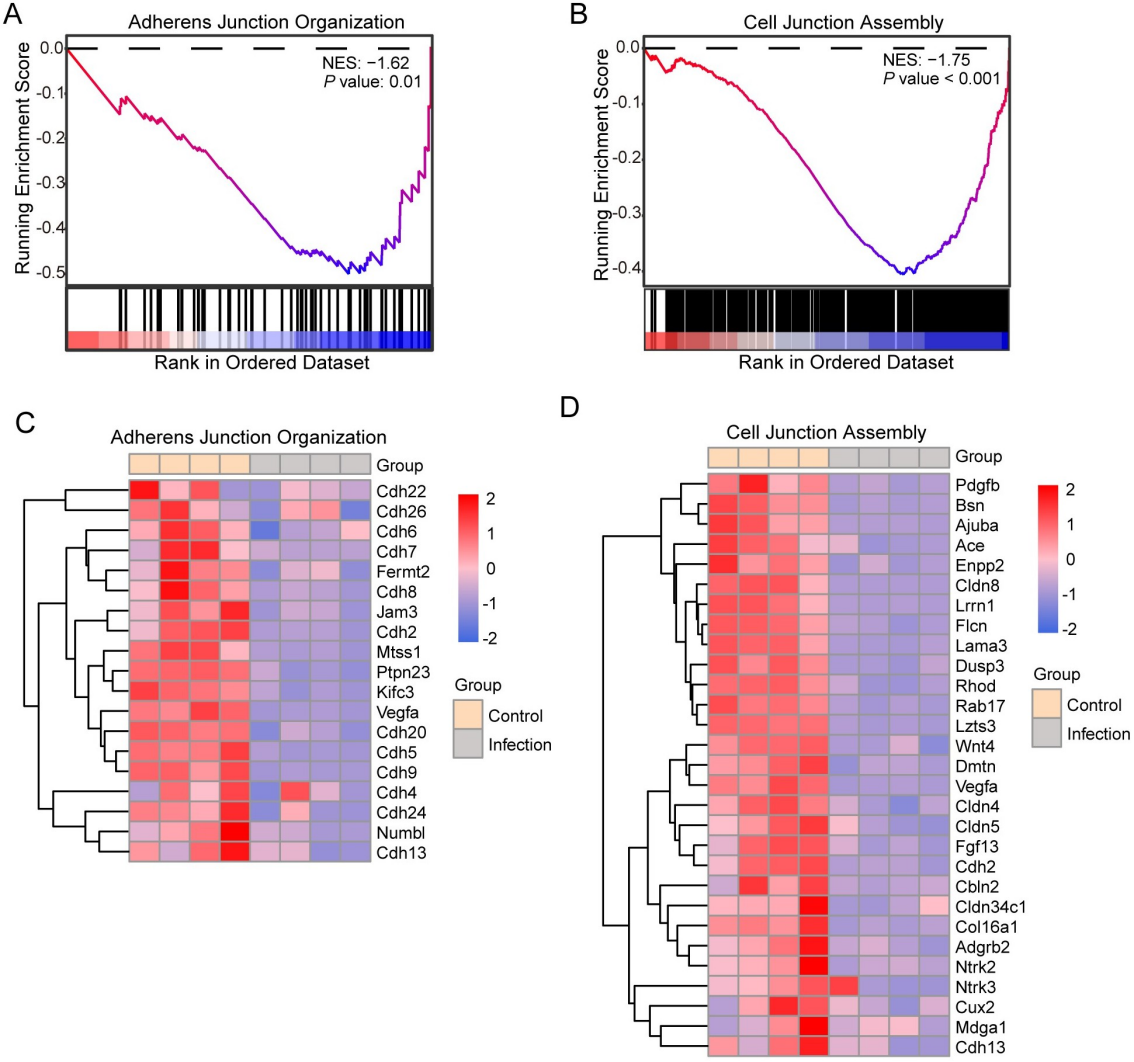
Upregulated of cell adhesion molecules and downregulated of adherens junction and cell junction molecules after *C*. *parvum* IId infection. The GSEA plot of the transcriptomic activation of adherens junction organization (A) and cell junction assembly (B) in *C*. *parvum* IId infected mice at 11 dpi. NES, normalized enrichment score. Black bars underneath the graph presented the rank positions of genes from the gene set. Heatmap of the transcriptomic activation of adherens junction organization (C) and cell junction assembly (D) in *C*. *parvum* IId infected and uninfected mice at 11 dpi. n = 4 independent samples. The color key (from blue to red) of abundance value indicated low to high expression levels.

To investigate the factors related to intestinal permeability, we focused on the cell junction molecules. We further analyzed the adherens junction organization and cell junction assembly molecules using gene set enrichment analysis (GSEA). The GSEA analysis performed on these two gene sets revealed a decrease in the expression of adherens junction genes and cell junction genes (Fig 5A and 5B). The heatmap showed a decrease in transcriptomic expression that affected the adherens junction organization in *C*. *parvum* IId infected mice at 11 dpi (Fig 5C). Most of the downregulated genes in this set were cadherins (CDH), indicating that various cadherins were involved in maintaining the epithelial barrier function and the cell-cell junctions were disassembled in epithelial cells after *C*. *parvum* infection. Subsequently, the heatmap revealed that the molecules responsible for cell junction assembly were also transcriptionally repressed in *C*. *parvum* IId infected mice at 11 dpi (Fig 5D). This set of downregulated genes also included several *CDH* genes and some genes (e.g. *cdh2*, *cdh13*) were significantly involved in both types. In addition, within the set of the cell junction assembly, the expression of several claudin (CLDN) genes, such as *cldn8*, *cldn4* and *cldn5*, were found to be downregulated after parasite infection, suggesting that the formation and function of tight junctions were disrupted in ileum cells.

### Transcription and protein level of E-cadherin in mouse ileum during *C*. *parvum* IId infection

**Fig 6:**
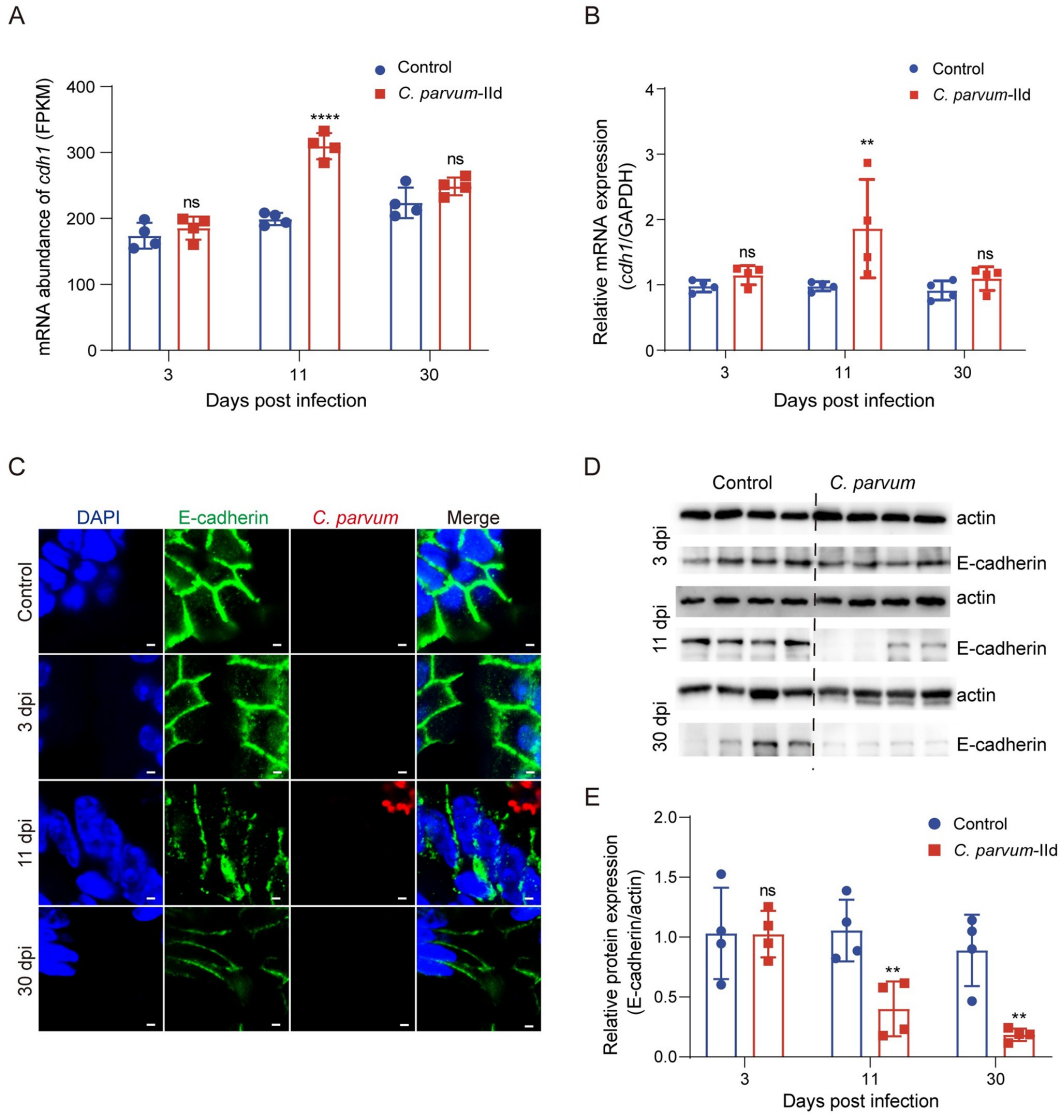
Transcription and expression of E-cadherin in mouse ileum during *C*. *parvum* IId infection. (A) Transcription of *cdh1* gene in *C*. *parvum* IId infected and control group were quantified by RNA-seq at indicated days post-infection. All data were represented as mean ± S.D. and analyzed by two-way ANOVA following Sidak’s method. ****, *P* < 0.0001; ns, not significant. (B) Transcription level of *cdh1* in *C*. *parvum* IId infected and control group was quantified by qPCR at indicated days post-infection. All data were represented as mean ± S.D. and analyzed by two-way ANOVA following Sidak’s method. **, *P* = 0.0042; ns, not significant. (C) Immunofluorescence of E-cadherin and *C*. *parvum* in mouse intestine. E-cadherin was stained with anti-E-cadherin antibodies (green), *C*. *parvum* was stained with Sporo-Glo (red) and nuclei were stained with DAPI (blue). Scale bars = 2 μm. (D) Western bolt of E-cadherin in mouse ileum. The mouse ileum samples were collected and lysed from *C*. *parvum* IId infected mice or control mice at indicated days post-infection. Proteins were transferred on the membranes and probed with E-cadherin and actin antibody. (E) Quantification and statistic of the E-cadherin/actin band intensities. Intensities of the bands corresponding to E-cadherin/actin were measured by Image-J. Densitometric analysis of relative band intensities with actin as internal control. All data are expressed as mean ± S.D., n = 4. Differences between *C*. *parvum* IId and control groups were analyzed using two-way ANOVA following Sidak’s method. **, *P* = 0.0060 at 11 dpi; **, *P* = 0.0032 at 30 dpi; ns, not significant.

Cellular tight junctions act as barriers to the electrochemical gradients required for intracellular ion transport, while cellular adhesion junctions function primarily function as mechanical junctions between neighboring cells [21]. E-cadherin is a protein that plays a crucial role in cell adhesion and has been linked to an increased risk of cancer metastasis. Although the GSEA analysis of downregulated molecules related to cell adherens junction and cell junction assembly did not include E-cadherin, the KEGG analysis of the data at 30 dpi suggested a potential risk of cancer metastasis after *C*. *parvum* IId infection (S3B Fig). Surprisingly, the transcriptional level of *cdh1*, which encodes E-cadherin, remained unchanged at 3 dpi and 30 dpi, and it was upregulated at 11 dpi (Fig 6A and 6B). The further analysis about the protein levels of E-cadherin in mouse ileum were measured by IFA. Changes in E-cadherin expression were observed after *C*. *parvum* IId infection, with the most significant changes occurring at 11 dpi (Fig 6C). At 11 dpi and 30 dpi, the fluorescence signal became lighter, and fluorescence lines were disrupted and disappeared compared to the control group (Fig 6C). The protein levels of E-cadherin were quantified by Western blot analysis. There was no significant difference in the protein level of E-cadherin in the mouse ileum after infection with *C*. *parvum* IId for 3 days (Fig 6D and 6E). However, a significant decrease in the E-cadherin expression was observed in the mouse ileum after infection with the *C*. *parvum* IId group for 11 and 30 days (Fig 6D and 6E). These results indicate that epithelial adherens junctions were disrupted during the parasite infection, which was mediated by E-cadherin.

## Discussion

This study conducted a preliminary investigation into the mechanism of diarrhea caused by *C*. *parvum* IId infection in neonatal mice. The findings suggest that *C*. *parvum* IId has a longer infection period than *C*. *parvum* IIa in C57BL6/j neonatal mice. The diarrhea disease severity was found to be higher in *C*. *parvum* IId than in *C*. *parvum* IIa from 6 dpi to 12 dpi. Furthermore, *C*. *parvum* IId caused significant damage to the intestinal epithelial cells. To investigate the mechanism of diarrhea in neonatal mice, we collected infected mouse ileums and analyzed their transcriptomes during the early (i.e. 3 dpi), peak (i.e. 11 dpi), and late (i.e. 30 dpi) periods. The results revealed that the upregulated DEGs mainly were mainly associated with the allograft and cytokine-cytokine receptor interaction pathways, while the downregulated DEGs were mainly associated with the lysosome and other glycan degradation pathways at 11 dpi. To explore the factors that caused the rise in intestinal permeability during parasite infection, we focused on the cell junction molecules. The analysis revealed that the adherens junction and cell junction pathways were downregulated at 11 dpi. In addition, the protein levels of E-cadherin, a crucial gene involved in the intestinal barrier, were significantly reduced at 11 dpi and 30 dpi.

Both *C*. *parvum* IIa and IId were able to infect neonatal mice, with the IId subtype exhibiting higher virulence than IIa. In this study, we used neonatal mice as the animal model and demonstrated both *C*. *parvum* IIa and IId could successfully infect 4-day-old mice. It is worth noting that the oocyst shedding period of *C*. *parvum* IId infection in neonatal mice was longer than that of *C*. *parvum* IIa infection. The peak oocyst shedding of IId infection was greater than 1.5×10^7^ OPG, whereas the peak oocyst shedding of IIa subtype infection was only 2.9×10^6^. Previous studies in China have shown that the IId subtype of *C*. *parvum* is a susceptible strain to dairy cows [22] and has been identified as the cause of severe diarrhea and high mortality in dairy cattle throughout the country [23]. However, there were only a few laboratory studies on the *C*. *parvum* IId [14,24]. A previous study also demonstrated that the oocyst shedding of *C*. *parvum* IId in interferon-γ-knockout mice was higher than that of *C*. *parvum* IIa, and the peak shedding window could persist for at least 50 days (end time point in that paper) [14]. In that paper, they chose the interferon-γ-knockout mouse as the experimental animal which is a suitable infection model for *C*. *parvum* infection [14]. However, it should be noted that interferon-γ-knockout mouse model may not fully reflect the infection patterns in young animals. In this study, we used neonatal mice as our experimental animal. We measured the oocyst shedding curve of mice were infected with *C*. *parvum* IIa or IId, then analyzed the dynamic changes of infection, injury and recovery. The ratio of intestinal villi to crypts decreased in mice heavily infected with *C*. *parvum* IId. Additionally, *C*. *parvum* IId infection caused more severe pathological damage in mice than *C*. *parvum* IIa. PAS staining revealed a significant reduction in mucin levels in the ileum of *C*. *parvum* IId infected mice at 11 dpi. These findings suggest that the intestinal epithelial barrier serves as the primary defense against pathogens. The barrier’s function was primarily impacted by the degradation of the epithelial junctional complexes, including tight junctions and adherens junctions [25]. Transmission electron micrographs revealed a significant disruption in the connections between intestinal epithelial cells in *C*. *parvum* IId infected mice at 11 dpi. The results of intestinal permeability tests and the pathological changes observed in the ileum suggest that the *C*. *parvum* IId is more virulent than the *C*. *parvum* IIa in the neonatal mice infection model.

We then explored the mechanism of diarrhea caused by *C*. *parvum* in neonatal mice by RNA-seq. Our analysis of differential gene expression identified 3375 DEGs and 511 DEGs in mouse ileal cells at 11 dpi and 30 dpi. Further analysis of KEGG and GO terms revealed that the cytokine-cytokine receptor interaction signaling pathway was significantly activated during the peak infection period. Previous studies have also confirmed that host innate immunity plays a significant role in the course of *C*. *parvum* infection [26]. In this study, KEGG analysis of the transcriptomes showed that the cell adhesion pathway was the second significantly upregulated in mice during *C*. *parvum* IId infection, which also seen in *C*. *parvum* infected HCT-8 cells [24,27]. There were some cases that excessive inflammatory response could result the host’s diarrheal death [28], and cell adhesion molecules were involved in regulating cell migration and apoptosis [29]. *C*. *parvum* is an obligatory parasite that doesn’t reside inside cells throughout its entire life cycle. During egress and re-invasion, it can cause significant damage to parasitic intestinal epithelial cells [30,31]. Therefore, the damage to intestinal epithelial cells could be raised from apoptosis and inflammatory response stemming from *C*. *parvum* infection [32,33].

The transcriptomic activation heatmap revealed that during *C*. *parvum* IId infection in mice, adherens junction and cell junction were downregulated. Additionally, the cadherin family was the most downregulated adherens junction genes. Cadherins are critical in maintaining normal tissue structure and morphology, and their downregulated expression is often associated with disease pathology such as tissue dysplasia, tumor formation, and metastasis [34]. Our research suggests that intestinal epithelial cells remain susceptible to cancer metastasis, even after recovering from *C*. *parvum* infection (i.e. 30 dpi). Previous studies showed the expression of occludin was downregulated in C57BL6/j mice infected with *C*. *parvum* for 24 and 48 hours [35]. Further research is needed to better understand the mechanisms of adherens junctions and cell junction molecules.

The E-cadherin protein is a calcium-dependent intercellular adhesion molecule and tumor suppressor protein. It plays a crucial role in maintaining epithelial intercellular adhesion [36]. Studies have shown that *C*. *parvum* infection in Caco-2 cells leads to downregulation of E-cadherin protein expression at both the mRNA and protein levels. This downregulation may cause dysfunction of the host intestinal epithelial barrier dysfunction and increased cell permeability [35]. However, our study found that the mRNA level of E-cadherin (gene: *cdh1*) was upregulated at 11 dpi. We conducted a more detailed analysis of E-cadherin specifically in intestinal epithelial cells using IFA and Western blot. The results indicated that protein levels of E-cadherin were significantly downregulated at 11 dpi and 30 dpi. Although the mechanism behind the downregulation of E-cadherin protein levels is not fully understood, there may be other pathways that contribute to its degradation. Further study is required to understand the inhibition of E-cadherin by *C*. *parvum* IId infection.

Our studies revealed that neonatal mice are susceptible to *C*. *parvum* IId, which causes a persistent high infection and severe pathological damage. At the peak parasite burden, the mice experienced a decrease in intestinal digestion and an increase in intestinal permeability. Transcriptome data indicated that multiple pathways were involved in the regulation of *C*. *parvum* infection. In this study, we also observed a significant upregulation of the inflammatory response in neonatal mice following *C*. *parvum* IId infection, as evidenced by transcriptomic analysis. However, we did not pursue further investigation into the potential association between the inflammatory process and the damage of mouse intestine. Then, we found out adherens junction and cell junction genes were downregulated during peak infection time. Finally, we demonstrated that the protein level of E-cadherin was downregulated during the peak and recovery infection periods.

## Supporting information

S1 Fig Pathogenicity of neonatal mice infected with *C*. *parvum* IIa, IId or uninfected control at indicated days post-infection. The 4-day-old C57BL/6j mice were infected with 1×10^5^ *C*. *parvum* IIa or IId strain per mouse. (A) The body weight of the mouse was measured every three days from 3 dpi to 30 dpi. (B) Diarrheal score of the mouse according to the fecal consistency from 3 dpi to 30 dpi.
(TIF)

S2 Fig Histological damage of neonatal mice infected with *C*. *parvum* IIa or IId. (A) H&E staining of the ileum of neonatal mice infected with *C*. *parvum* IIa, IId or uninfected control at indicated days post-infection. Scale bars = 2 μm. (B) PAS staining of the mouse ileum at indicated days post-infection. Red arrows point to lymphocytes, green arrow points to eosinophilic granulocyte; scale bars = 20 μm.
(TIF)

S3 Fig Analysis of the KEGG pathway in *C*. *parvum* IId infected and uninfected mice at 30 dpi. (A) The KEGG enrichment of upregulated genes in *C*. *parvum* IId infected mice compared with uninfected mice at 30 dpi. (B) The KEGG enrichment of downregulated genes in *C*. *parvum* IId infected mice compared with uninfected mice at 30 dpi.
(TIF)

S4 Fig Patterns of gene expressions in *C*. *parvum* IId infected and uninfected mice at indicated days post-infection. (A) The profiles of DEGs during the infections. Profiles ordered based on the *P*-value significance of number of genes assigned versus expected. (B) The KEGG enrichment of profiles 11, profiles 10, profiles 12 and profiles 16.
(TIF)
