# Supplementary figures and images for: Cryptosporidium parvum disrupts intestinal epithelial barrier in neonatal mice through downregulation of cell junction molecules
Source: PLoS Negl Trop Dis. 2024 May 24;18(5):e0012212. doi: 10.1371/journal.pntd.0012212 (PMC11156435; doi:10.1371/journal.pntd.0012212)

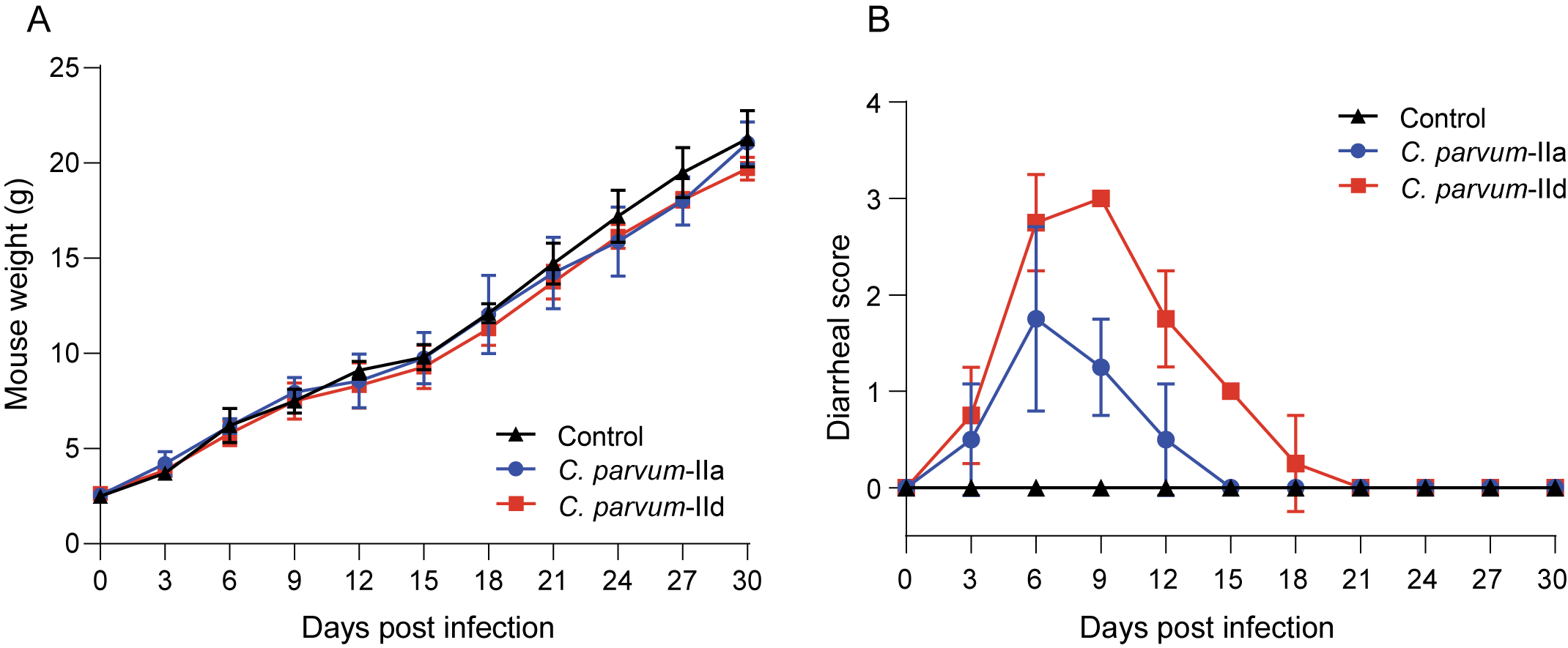

Supplement: S1 Fig — The 4-day-old C57BL/6j mice were infected with 1×10^5^ C. parvum IIa or IId strain per mouse. (A) The body weight of the mouse was measured every three days from 3 dpi to 30 dpi. (B) Diarrheal score of the mouse according to the fecal consistency from 3 dpi to 30 dpi. (TIF) [file pntd.0012212.s001.tif]

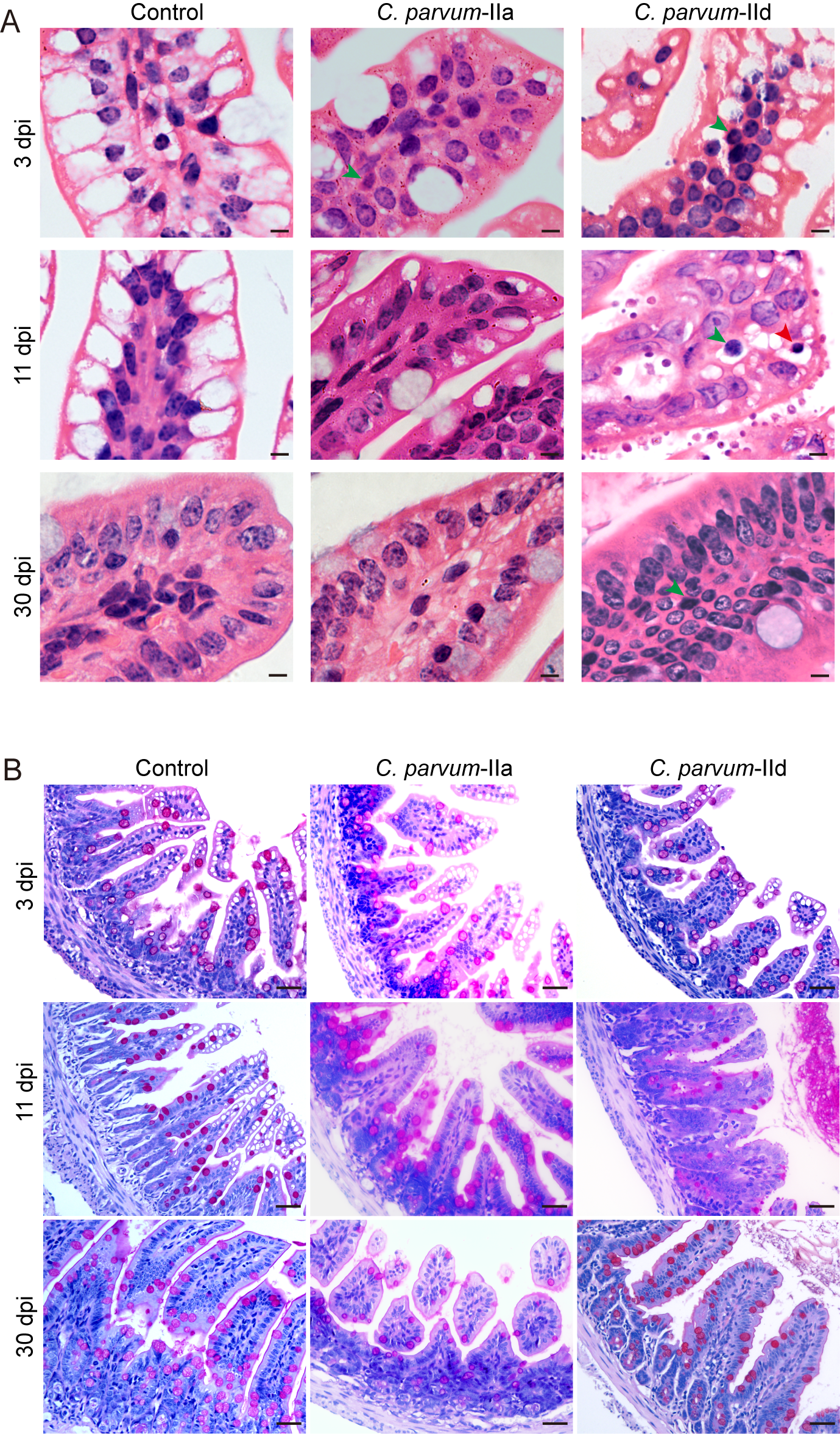

Supplement: S2 Fig — (A) H&E staining of the ileum of neonatal mice infected with C. parvum IIa, IId or uninfected control at indicated days post-infection. Scale bars = 2 μm. (B) PAS staining of the mouse ileum at indicated days post-infection. Red arrows point to lymphocytes, green arrow points to eosinophilic granulocyte; scale bars = 20 μm. (TIF) [file pntd.0012212.s002.tif]

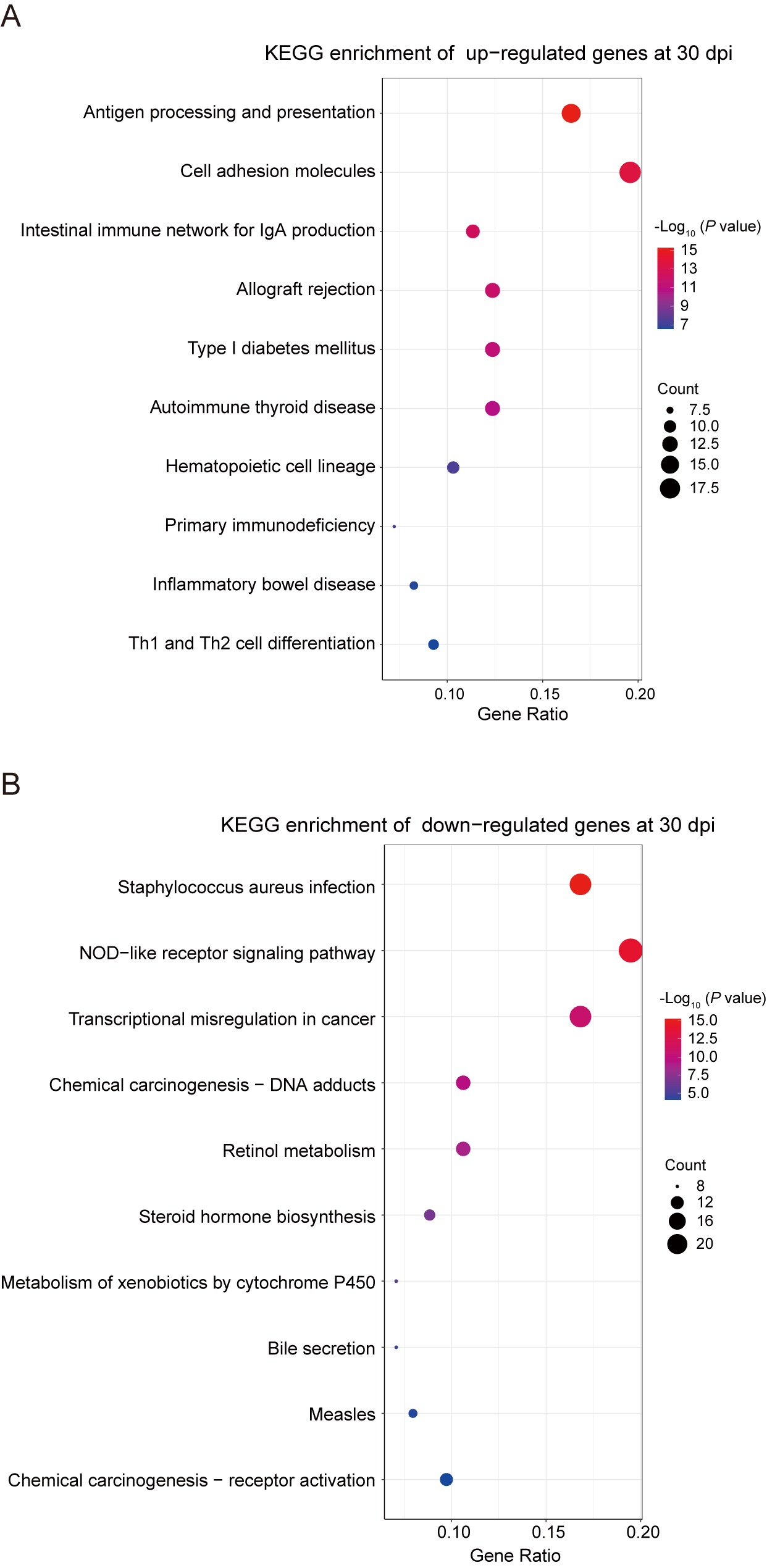

Supplement: S3 Fig — (A) The KEGG enrichment of upregulated genes in C. parvum IId infected mice compared with uninfected mice at 30 dpi. (B) The KEGG enrichment of downregulated genes in C. parvum IId infected mice compared with uninfected mice at 30 dpi. (TIF) [file pntd.0012212.s003.tif]

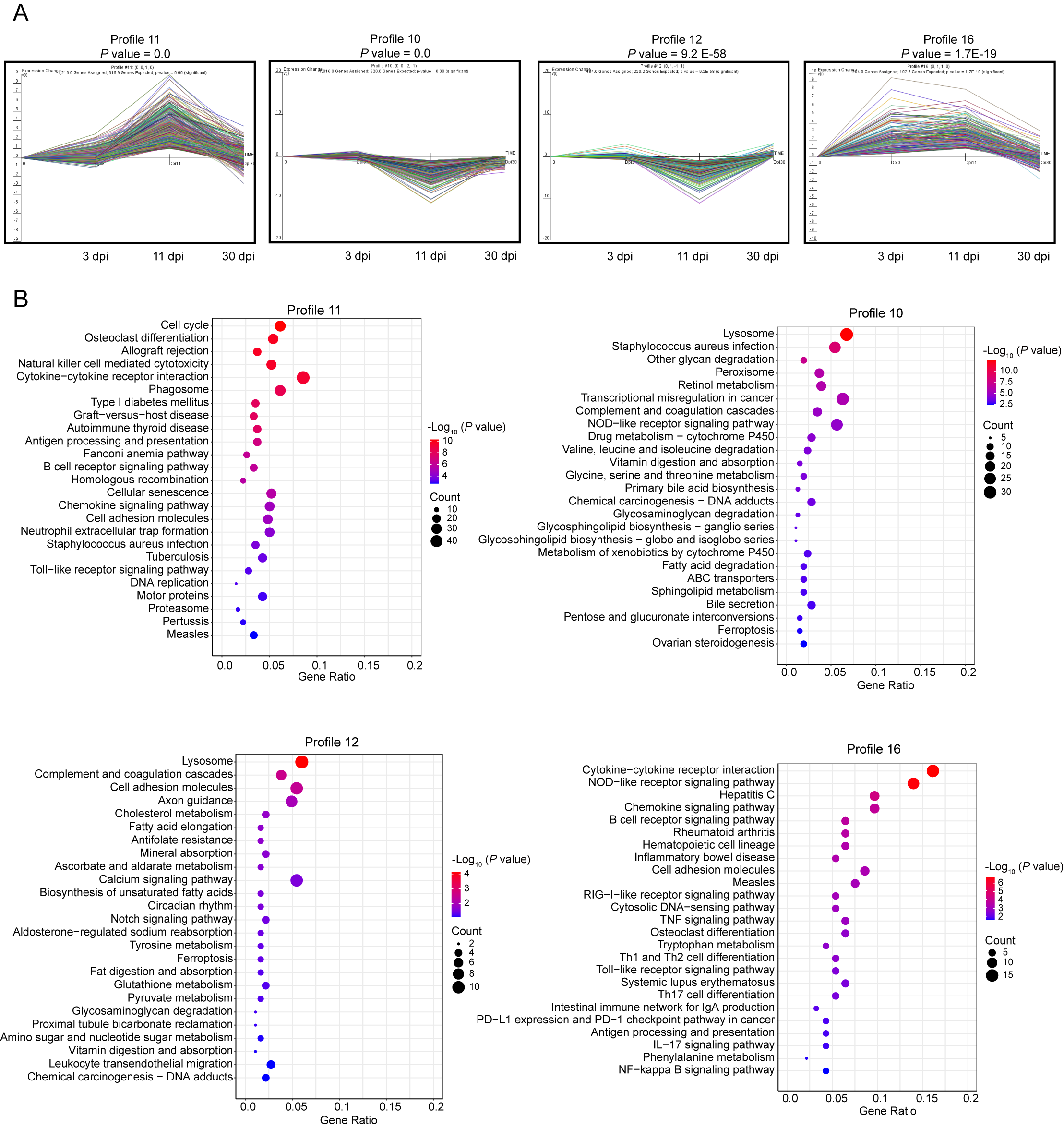

Supplement: S4 Fig — (A) The profiles of DEGs during the infections. Profiles ordered based on the P-value significance of number of genes assigned versus expected. (B) The KEGG enrichment of profiles 11, profiles 10, profiles 12 and profiles 16. (TIF) [file pntd.0012212.s004.tif]
